# Effect of annual hospital admissions of out-of-hospital cardiac arrest patients on prognosis following cardiac arrest

**DOI:** 10.1186/s12873-022-00685-7

**Published:** 2022-07-07

**Authors:** Takumi Tsuchida, Kota Ono, Kunihiko Maekawa, Mariko Hayamizu, Mineji Hayakawa

**Affiliations:** 1 Department of Emergency Medicine, Hokkaido University Hospital, N14W5 Kita-ku, Sapporo, 060-8648 Japan; 2 Ono Biostat Consulting, Narita-higashi, Suginami-ku, Tokyo, 166-0015 Japan

**Keywords:** Cardiopulmonary resuscitation, Hospital volume, Neurological outcome, Out-of-hospital cardiac arrest, Prediction, Prognosis

## Abstract

**Background:**

Although the prognosis of patients treated at specialized facilities has improved, the relationship between the number of patients treated at hospitals and prognosis is controversial and lacks constancy in those with out-of-hospital cardiac arrest (OHCA). This study aimed to clarify the effect of annual hospital admissions on the prognosis of adult patients with OHCA by analyzing a large cohort.

**Methods:**

The effect of annual hospital admissions on patient prognosis was analyzed retrospectively using data from the Japanese Association for Acute Medicine OHCA registry, a nationwide multihospital prospective database. This study analyzed 3632 of 35,754 patients hospitalized for OHCA of cardiac origin at 86 hospitals. The hospitals were divided into tertiles based on the volume of annual admissions. The effect of hospital volume on prognosis was analyzed using logistic regression analysis with multiple imputation. Furthermore, three subgroup analyses were performed for patients with return of spontaneous circulation (ROSC) before arrival at the emergency department, patients admitted to critical care medical centers, and patients admitted to extracorporeal membrane oxygenation-capable hospitals.

**Results:**

Favorable neurological outcomes 30 days after OHCA for patients overall showed no advantage for medium- and high-volume centers over low-volume centers; Odds ratio (OR) 0.989, (95% Confidence interval [CI] 0.562-1.741), OR 1.504 (95% CI 0.919-2.463), respectively. However, the frequency of favorable neurological outcomes in OHCA patients with ROSC before arrival at the emergency department at high-volume centers was higher than those at low-volume centers (OR 1.955, 95% CI 1.033-3.851).

**Conclusion:**

Hospital volume did not significantly affect the prognosis of adult patients with OHCA. However, transport to a high-volume hospital may improve the neurological prognosis in OHCA patients with ROSC before arrival at the emergency department.

**Supplementary Information:**

The online version contains supplementary material available at 10.1186/s12873-022-00685-7.

## Background

Out-of-hospital cardiac arrest (OHCA) occurs in 250,000 to 300,000 patients worldwide each year [1]. Advances have been made in the management of cardiac arrest, including modern cardiopulmonary resuscitation (CPR), extracorporeal CPR, emergency cardiovascular therapy, and targeted temperature management [2]. However, the in-hospital survival and neurologically intact survival rates remain disappointingly low in patients with a successful return of spontaneous circulation (ROSC) [3].

The outcomes for patients with OHCA have been shown to improve with the quality of round-the-clock post-resuscitation care [4, 5], while the frequency of post-resuscitation care in the emergency department also exerts a positive impact on patient outcomes [6, 7]. Current European resuscitation guidelines also indicate that transport of OHCA patients to high-volume centres may improve prognosis [8, 9]. However, there is a coexistence of studies showing improved outcomes in hospitals with larger volume [10–14], and studies showing no improvement [15–17]. One conclusion drawn today from low-quality data is that cardiac arrest centers may be associated with improved outcomes at discharge, but the certainty of that evidence is very low [18].

The relationship between the number of surgeries performed in hospitals and patient outcomes has been studied since the 1980s [19, 20]. In recent years, several studies have reported on the relationship between patient outcomes and medical services rendered by hospitals and physicians in various fields, not limited to surgery [21]. Specialized clinical departments such as stroke and coronary artery care units were established prior to the establishment of specialized treatment facilities for patients with cardiac arrest, and the effect of these facilities on prognosis improvement has been proven [22, 23].

The prognosis of the OHCA of non-cardiac origin is extremely poor and our interest is in the improvemnt of prognosis of OHCA of cardiac origin. Therefore, this study was limited to cardiogenic OHCA. In this study, we evaluated the impact of the volume of annual hospital admissions on the prognosis of patients with OHCA using a large cohort from a nationwide study.

## Methods

### Participants/data source

This study conducted a post-hoc analysis of patients included in the Japanese Association for Acute Medicine OHCA (JAAM-OHCA) registry. This database is a nationwide multihospital prospective registry of hospital data collected according to the Utstein template, and in-hospital data, including treatments, arterial blood gas levels, and outcomes [24].

### Setting

All Japanese emergency medical services (EMS) personnel can perform CPR in accordance with the Japanese resuscitation guidelines, which are based on the statement of the International Liaison Committee on Resuscitation. EMS personnel are legally prohibited from terminating resuscitation at the scene, and all patients with OHCA are transported to the hospital unless death is certain. The destination is usually not altered due to the cause of cardiac arrest. EMS usually transport patients with OHCA to the nearest emergency hospital, which is under the purview of the local medical control transports some cases, patients with ROSC may be transferred to a hospital that can provide more advanced care. In this registry, patient information is recorded when the hospital that first admitted the patient is a participating research hospital, and information on the prognosis is provided to the research facility by the transferring hospital. This registry includes all patients with OHCA, irrespective of internal or external causes. We used the JAAM-OHCA registry data for patients admitted between June 2014 and September 2017. Eighty-six hospitals and 35,754 patients were registered during this period.

### Patients

The following cases were excluded from the analysis in this study were patients: (a) aged < 17 years, (b) with unknown initial rhythm, (c) who experienced ROSC upon contact with the EMS, (d) with an unknown prognosis 30 days after cardiac arrest, (e) with extrinsic cardiac arrest, (f) with cardiac arrest due to other medical causes, and (g) who died in the emergency department.

### Outcomes and definitions

The primary outcome in this study was the neurological outcome 30 days after cardiac arrest, and the secondary outcome was survival 30 days after cardiac arrest. Neurological outcomes were evaluated using the cerebral performance category (CPC) scale [25]. Patients with a score of CPC 1 or CPC 2 were designated as having a favorable neurological outcome. Patients’ prognosis 30 days after cardiac arrest was obtained from their current condition if they were still hospitalized, or from telephone or written survey responses from registered facilities if they had been transferred or discharged from the hospital.

### Study design

This retrospective analysis was conducted using a prospective registry (JAAM-OHCA registry).

### Statistical analysis

Hospitals were divided into three equal groups according to the number of patients with OHCA of cardiac origin (i.e., patient volume) received per year. This classification of groups was based on the number of patients admitted after OHCA of cardiac origin. In the present study, patient volume was equally divided by the number of hospitals, resulting in an unequal number of patients in each group. We selected the following potential patient-related factors that may affect the prognosis: sex, age, contact between doctor and patient before arrival at hospital, motor score on the Glasgow coma scale upon arrival at the emergency department (ED), defibrillation performed by EMS, use of airway devices by EMS, types of airway devices used by the EMS, primary electrocardiography rhythm at the scene, witness by bystander, CPR initiated by a bystander, defibrillation performed by a bystander, intravenous fluid administration by EMS, dosage of adrenaline administered until arrival at the ED, presence of ROSC prior to arrival at the hospital, presence of ROSC on arrival at the hospital, time from calling the EMS to arrival at the scene, time from arrival at the scene to arrival at the ED, and laboratory data on arrival at the ED (serum urea nitrogen, serum creatinine, serum total protein, serum albumin, pH, partial pressure of carbon dioxide, partial pressure of oxygen, HCO_3_, base excess, lactate, and glucose). These variables were used for multiple imputation and generalized estimating equations described later in this section.

We also investigated the following four subgroups: patient with ROSC prior to arrival at hospital, patient with ROSC prior to arrival at hospital, patients who were transported to critical-care medical centers, and patients who were transported to ECMO-capable hospitals. The same outcomes, potential patient factors, and hospital-volume categories were used as those for the main population (patients with OHCA).

We presented the patient and hospital characteristics of the three tertiles of hospital volume (low, middle and high). Continuous variables were presented as medians with interquartile ranges and categorical variables were presented as numbers and percentages. The Jonckheere-Terpstra test and Cochran-Armitage trend test were used for continuous and categorical variables, respectively. Chi-square test was used for categorical variables with multi-category. We employed multiple imputation by chained equations to address missing data assuming the missing mechanism as missing at random, and 100 imputed datasets were created. Missing potential patient-related factors were imputed and the numbers of missing data were shown in a table of characteristics of patients. We performed generalized estimating equations to account for the clustering of patients within each hospital and to examine the association between hospital volume and survival 30 days after cardiac arrest or rehabilitation 30 days after cardiac arrest, adjusting for the above-mentioned patient factors except for pH, partial pressure of carbon dioxide and HCO3 since these variables were highly correlated with base excess. We also assessed linear trend by performing a trend test using contrasts of coefficients obtained from generalized estimating equations. Odds ratios and 95% confidence intervals (CIs) were calculated. All analyses were performed using R version 3.6.3. All reported *p*-values were two-tailed, and differences with p-values (p) < 0.05 were considered statistically significant.

## Results

### Flow of patients enrollments

**Fig. 1:**
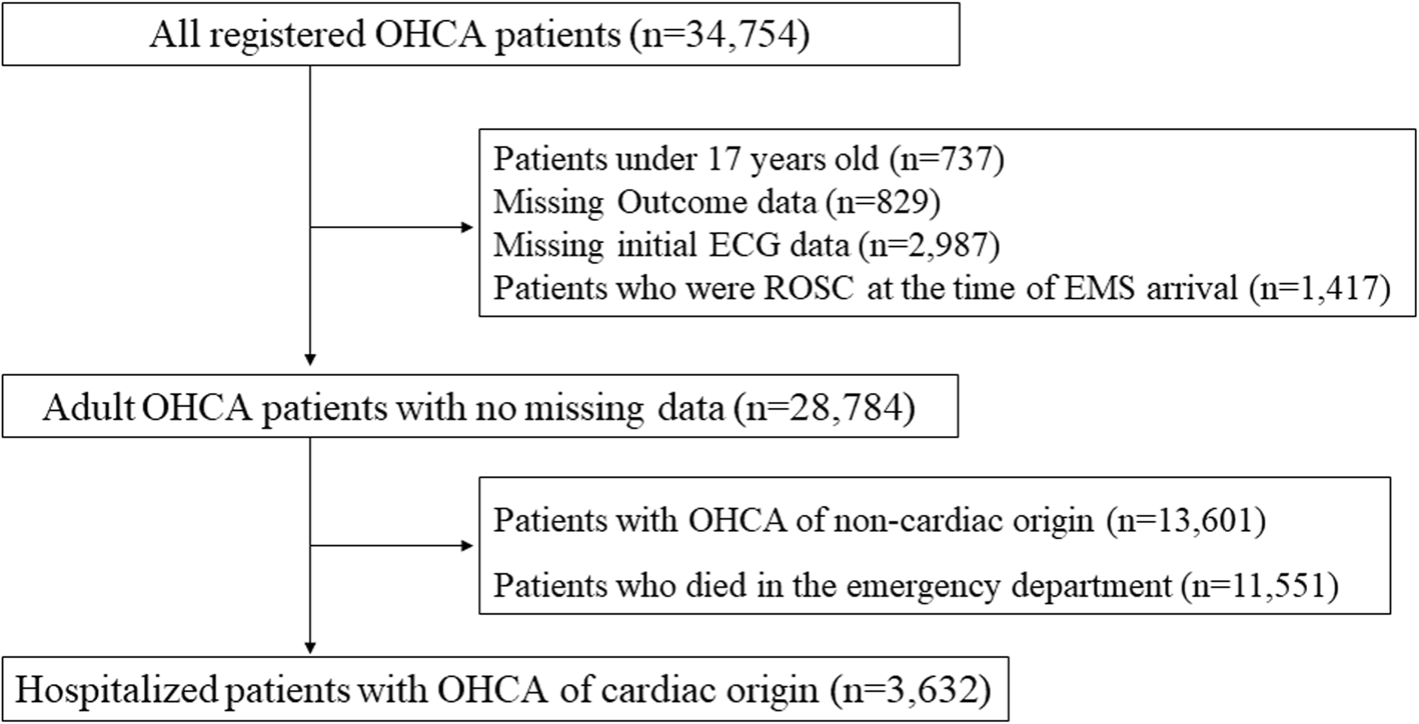
Flow chart of patient enrollment in this study. The *n* in the figure indicates the number of patients. OHCA: out-of-hospital cardiac arrest, ECG: electrocardiography, ROSC: return of spontaneous circulation, EMS: emergency medical services

Adult patients with OHCA (*n* = 28,784) were retrieved from those enrolled in the JAAM-OHCA registry (*n* = 34,754). Patients under 17 years of age (*n* = 737), patients with missing data (*n* = 3816), and patients who had already attained ROSC at the time of EMS contact (*n* = 1417) were excluded. The current study also excluded patients with non-cardiac causes of OHCA (*n* = 13,601) and patients who died in the emergency room (*n* = 11,551), to ensure accurate assessment of the impact of the volume of annual hospital admissions on OHCA patients. Finally, the remaining 3632 patients with OHCA of cardiac origin were included in the analysis (Fig. 1).

### Characteristics of hospitals and patients

Twenty-nine hospitals (250 patients) were categorized as low-volume, 28 hospitals (817 patients) were categorized as medium volume, and 29 hospitals (2565 patients) were categorized as high volume based on the number of patients admitted after OHCA of cardiac origin. The characteristics of the hospitals are presented in Table 1. The percentage of critical-care medical centers and number of doctors on the night/holiday shift were positively correlated with hospital volume (*p* < 0.001 and 0.018, respectively). Table 2 shows the characteristics of the patients included in the analysis based on hospital volume. Most characteristics related to the hospitals and patient transport did not differ significantly among the hospital-volume categories. However, the number of patients for whom physician care had been initiated prior to hospital arrival and the number of patients who required extracorporeal cardiopulmonary resuscitation (E-CPR) had higher percentages in high-volume hospital.

**Table 1:** Characteristic of the hospitals in each group

	Low-Volume Hospital	Middle-Volume Hospital	High-Volume Hospital	*P*-value
Institutions, n	29	28	29	
Number of beds	604.0 (460.0–800.0)	624.0 (470.8–758.2)	605.0 (520.0–768.0)	0.712
Number of ICU beds	10.0 (6.0–14.0)	9.5 (6.0–18.5)	12.0 (8.0–19.0)	0.152
Critical care medical center	16 (55.2%)	21 (75.0%)	29 (100.0%)	< 0.001
Number of all OHCA patients delivered in the last 1 year	70.0 (31.0–106.0)	123.5 (95.0–150.0)	230.0 (158.0–320.0)	< 0.001
Number of hospitalized OHCA of cardiac origin patients per year	4.0 (2.2-5.0)	10.7 (9.4-12.0)	22.8 (18.5-32.1)	< 0.001
Number of doctors in the treatment of cardiac arrest cases at emergency department				
Day shift				0.210
One doctor, n (%)	2 (6.9%)	0 (0.0%)	1 (3.4%)	
Two doctors, n (%)	6 (20.7%)	2 (7.1%)	2 (6.9%)	
≥ 3 doctors, n (%)	21 (72.4%)	26 (92.9%)	26 (89.7%)	
Night/holiday shift				0.018
One doctor, n (%)	6 (20.7%)	1 (3.6%)	1 (3.4%)	
Two doctors, n (%)	13 (44.8%)	11 (39.3%)	6 (20.7%)	
≥ 3 doctors, n (%)	10 (34.5%)	16 (57.1%)	22 (75.9%)	
Number of nurses in the treatment of cardiac arrest cases at emergency department				0.379
Day shift				
One nurse, n (%)	2 (6.9%)	2 (7.1%)	6 (20.7%)	
Two nurses, n (%)	11 (37.9%)	11 (39.3%)	12 (41.4%)	
≥ 3 nurses, n (%)	16 (55.2%)	15 (53.8%)	11 (37.9%)	
Night/holiday shift				0.606
One nurse, n (%)	10 (34.5%)	6 (21.4%)	8 (27.6%)	
Two nurses, n (%)	10 (34.5%)	13 (46.4%)	15 (51.7%)	
≥ 3 nurses, n (%)	9 (31.0%)	9 (32.2%)	6 (20.7%)	
Availability of medical specialists in hospital				
Emergency Physician, n (%)	27 (93.1%)	27 (96.4%)	29 (100.0%)	0.152
Intensivist, n (%)	23 (79.3%)	20 (71.4%)	26 (89.7%)	0.323
Anesthesiologist, n (%)	25 (86.2%)	25 (89.3%)	21 (72.4%)	0.166
Cardiologist, n (%)	25 (86.2%)	27 (96.4%)	27 (93.1%)	0.337
ECMO-capable hospitals, n (%)	26 (89.7%)	27 (96.4%)	29 (100.0%)	0.061

Data are presented as the median (25th-75th percentile), percentage, or numbers

*ECMO* extra corporeal membrane oxygenation, *ICU* intensive care unit, *OHCA* out-of-hospital cardiac arrest

**Table 2:** Characteristics of patients with OHCA of cardiac origin in each group

	Low-Volume Hospital	Middle-Volume Hospital	High-Volume Hospital	*P*-value
Institutions, n	29	28	29	
Patients, n	250	817	2565	
^d^Male, n (%)	170 (68.0%) [0]	575 (70.4%) [0]	1852 (72.2%) [0]	0.106
^d^Age, year	70.0 (60.0–82.0) [0]	71.0 (60.0–82.0) [0]	69.0 (58.0–79.0) [0]	< 0.001
Cause of OHCA, n (%)	[0]	[0]	[0]	0.035
Acute coronary syndrome	77 (30.8%)	269 (32.9%)	805 (31.4%)	
Other cardiac^a^	75 (30.0%)	226 (27.7%)	620 (24.2%)	
Presumed cardiac	98 (39.2%)	322 (39.4%)	1140 (44.4%)	
^d^Witness by bystander, n (%)	171 (68.4%) [0]	564 (69.0%) [0]	1754 (68.4%) [0]	0.839
^d^CPR initiated by bystander, n (%)	130 (52.0%) [0]	358 (43.8%) [0]	1233 (48.1%) [0]	0.741
^d^Defibrillation by bystander, n (%)	5 (2.0%) [0]	48 (5.9%) [0]	160 (6.2%) [0]	0.025
^d^Primary ECG rhythm at the scene, n (%)	[0]	[0]	[0]	0.042
Ventricular fibrillation	89 (35.6%)	325 (39.8%)	1113 (43.4%)	
Pulseless ventricular tachycardia	1 (0.4%)	10 (1.2%)	16 (0.6%)	
Pulseless electrical activity	71 (28.4%)	223 (27.3%)	701 (27.3%)	
Asystole	89 (35.6%)	259 (31.7%)	735 (28.7%)	
Treatments by EMS				
^d^Defibrillation, n (%)	58 (23.2%) [0]	196 (24.0%) [0]	722 (28.1%) [0]	0.010
^d^Use of airway devices, n (%)	[0]	[0]	[0]	< 0.001
Bag valve mask	179 (71.6%)	485 (59.4%)	1041 (40.6%)	
Laryngeal mask airway	5 (2.0%)	21 (2.6%)	171 (6.7%)	
Esophageal obturator airway	53 (21.2%)	267 (32.7%)	940 (36.6%)	
Tracheal intubation	13 (5.2%)	44 (5.4%)	413 (16.1%)	
^d^Intravenous fluid administration, n (%)	78 (31.6%) [3]	329 (40.3%) [0]	1120 (43.7%) [0]	< 0.001
^d^Treatments by doctor before arrival at ED, n (%)	23 (9.2%) [0]	91 (11.1%) [0]	566 (22.1%) [0]	< 0.001
^d^Adrenaline dosage until arrival at ED (mg)	0.0 (0.0–2.0) [123]	1.0 (0.0–2.0) [290]	1.0 (1.0–3.0) [1183]	< 0.001
Time (min)				
^d^From calling EMS to arrival at the scene (min)	9.0 (7.0–11.0) [0]	8.0 (7.0–10.0) [0]	8.0 (6.0–10.0) [1]	< 0.001
^d^From arrival at the scene to arrival at the ED (min)	22.0 (17.0–29.0) [0]	22.0 (17.0–29.0) [3]	24.0 (18.0–31.0) [27]	< 0.001
ECG rhythm on arrival at ED, n (%)	[0]	[0]	[0]	< 0.001
Ventricular fibrillation	28 (11.2%)	101 (12.4%)	469 (18.3%)	
Pulseless ventricular tachycardia	5 (2.0%)	8 (1.0%)	15 (0.6%)	
Pulseless electrical activity	65 (26.0%)	220 (26.9%)	645 (25.1%)	
Asystole	87 (34.8%)	249 (30.5%)	714 (27.8%)	
Return of spontaneous circulation	65 (26.0%)	239 (29.3%)	722 (28.1%)	
Extracorporeal CPR, n (%)	37 (14.8%) [0]	144 (17.6%) [0]	667 (26.0%) [0]	< 0.001
Time from arrival at ED to start of VA-ECMO (min)	35.5 (26.8–63.0) [214]	40.5 (29.0–65.0) [673]	29.0 (20.0–41.0) [1902]	< 0.001
Laboratory data on arrival at the ED				
^d^Serum Urea nitrogen (mg/dl)	19.1 (14.1–30.8) [83]	19.0 (14.9–27.0) [314]	18.9 (14.0–26.6) [833]	0.131
^d^Serum Creatinine (mg/dl)	1.15 (0.95–1.60) [84]	1.13 (0.90–1.43) [316]	1.11 (0.90–1.50) [814]	0.616
^d^Serum total protein (g/dl)	6.2 (5.7–6.8) [90]	6.1 (5.5–6.7) [311]	6.1 (5.4–6.6) [902]	0.009
^d^Serum albumin (g/dl)	3.4 (3.0–3.8) [93]	3.4 (2.9–3.8) [332]	3.3 (2.8–3.7) [906]	< 0.001
^d^pH	7.06 (6.92–7.25) [89]	7.10 (6.93–7.25) [261]	7.06 (6.90–7.25) [755]	0.066
^d^PaCO_2_ (mmHg)	52.3 (38.6–77.4) [92]	52.5 (39.5–73.5) [261]	51.80 (37.3–77.5) [753]	0.863
^d^PaO_2_ (mmHg)	144.0 (82.9–288.7) [98]	137.5 (81.4–280.0) [261]	169.0 (82.6–340.0) [754]	0.012
^d^HCO3 (mEq/l)	16.2 (12.8–19.7) [96]	16.2 (12.1–19.8) [273]	15.4 (11.9–18.8) [756]	0.006
^d^Base excess (mEq/l)	−13.1 (−17.9–-7.3) [96]	−13.0 (−18.7–-7.0) [277]	−14.4 (−20.2–-8.6) [765]	< 0.001
^d^Lactate (mg/dl)	100.8 (72.0–129.6) [113]	90.8 (59.2–122.1) [306]	95.0 (65.7–128.7) [778]	0.187
^d^Glucose (mg/dl)	244.0 (172.25–305.25) [100]	260.5 (198.00–330.50) [279]	263.0 (199.0–330.0) [801]	0.230
^d^Patient with ROSC prior to arrival at ED, n (%)	65 (26.0%) [0]	239 (29.3%) [0]	722 (28.1%) [0]	0.854
^d^Patient with ROSC after arrival at ED, n (%)	64 (25.6%) [0]	246 (30.1%) [0]	757 (29.5%) [0]	0.439
Time from calling EMS to the first ROSC before arriving at the ED (min)^b^	22.0 (15.0-27.0) [177]	18.0 (13.0–25.0) [541]	19.0 (13.0–26.0) [1628]	0.749
Time from calling EMS to the first ROSC after arriving at the ED (min)^c^	44.0 (34.0–56.5) [80]	42.5 (34.0–57.0) [335]	44.0 (34.0–57.0) [1072]	0.852
Time from ED arrival to ROSC after admission (min)^c^	13.0 (8.0–20.0) [82]	13.0 (8.0–22.0) [335]	13.0 (8.0–24.0) [1074]	0.150
^d^Motor score of GCS in ED	1.0 (1.0-1.0) [0]	1.0 (1.0-1.0) [0]	1.0 (1.0-1.0) [0]	0.351
Therapeutic hypothermia, n (%)	74 (29.6%) [0]	256 (31.3%) [0]	955 (37.2%) [0]	< 0.001
Outcomes 30 days after cardiac arrest	[0]	[0]	[0]	
Survive, n (%)	81 (32.4%)	269 (32.9%)	883 (34.4%)	0.353
Favorable neurological outcome, n (%)	46 (18.4%)	167 (20.4%)	543 (21.2%)	0.308

Data are presented as the median (25th-75th percentile), percentage, or numbers

The number in “[]” indicates the number of missing measurements or patients not included in the analysis

*CPR* cardiopulmonary resuscitation, *ECG* electrocardiogram, *ED* emergency department, *EMS* emergency medical services, *GCS* Glasgow coma scale, *OHCA* out-of-hospital cardiac arrest, *ROSC* return of spontaneous circulation, *VA-ECMO* veno-arterial extra corporeal membrane oxygenation

^a^“Other cardiac” causes include heart failure, valvular disease, cardiomyopathy, and cardiac diseases other than identified acute coronary syndrome

^b^Data limited to cases with ROSC prior to ED arrival

^c^Data limited to cases with cardiac arrest on arrival at the ED

^d^Selected as potential patient-related factors

### Primary outcome (neurological outcome 30 days after cardiac arrest)

**Fig. 2:**
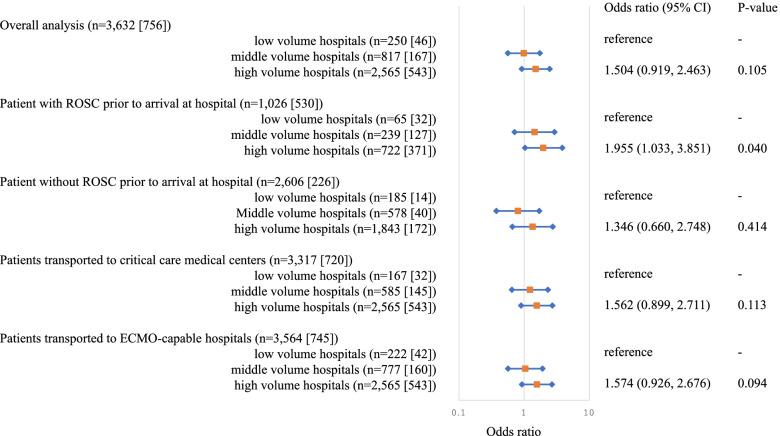
Adjusted odds ratio for favorable neurological outcomes 30 days after OHCA. Odds ratio of middle-volume hospitals and high-volume hospitals to low-volume hospital (reference) for favorable neurological outcomes 30 days after hospitalization. “n*”* in the figure indicates the number of patients. The number in “[]” represents the number of people who actually had a favorable neurological outcome. CI: confidence interval, ROSC: return of spontaneous circulation, ECMO: extracorporeal membrane oxygenation, OHCA: out-of-hospital cardiac arrest

Adjusted Odds ratio for favorable neurological outcomes 30 days after OHCA were presented in Fig. 2. In an analysis of the overall patients, there was no advantage of middle- and high-volume hospitals over low-volume hospitals (OR 0.989, 1.504, 95% CI 0.562-1.741, 0.919-2.463, respectively). A subgroup analysis focusing on patients transported to critical care medical centers also showed no advantage of middle- and high-volume hospitals over low-volume hospitals (OR 1.226, 1.562, 95% CI 0.649-2.316, 0.899-2.711, respectively). A subgroup analysis focused on patients transported to ECMO-capable hospitals showed the same results (OR 1.040, 1.574, 95% CI 0.565-1.913, 0.926-2.676, respectively). However, the frequency of favorable neurological outcomes in OHCA patients with ROSC before arrival at the emergency department at high-volume centers was higher than those at low-volume centers (OR 1.346, 95% CI 0.660-2.748). Conversely, the analysis for patients with OHCA who did not achieve ROSC before arrival at the ED showed no significant difference between neurological outcome and institutional volume (OR 0.800, 1.346, 95% CI 0.375-1.705, 0.660-2.748, respectively).

### Secondary outcome (survival 30 days after cardiac arrest)

**Fig. 3:**
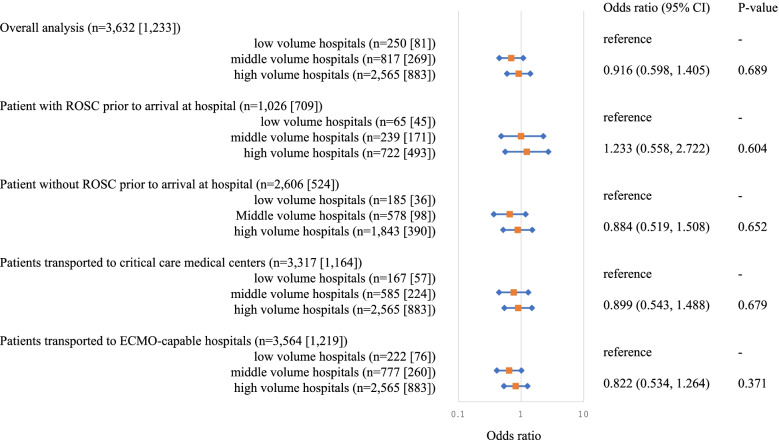
Adjusted odds ratio for 30-day survival after OHCA. Odds ratio of middle-volume hospitals and high-volume hospitals to low volume hospitals (reference) when the outcome is survival rate after 30 days of hospitalization. “n*”* in the figure indicates the number of patients. The number in “[]” represents the number of people who actually survived. CI: confidence interval, ROSC: return of spontaneous circulation, ECMO: extracorporeal membrane oxygenation, OHCA: out-of-hospital cardiac arrest

The same analysis was performed with Outcome as 30 days survival after OHCA, but no significant results were found in all subgroups (Fig. 3). The characteristics of OHCA patients in each subgroup are shown in Additional files 1, 2, 3 and 4.

## Discussion

This study retrospectively analyzed the effect of institutional volume on patient prognosis using data from 3632 adults hospitalized for OHCA of cardiac origin from more than 30,000 individuals registered with the nationwide OHCA registry. In particular, neurological outcomes tended to improve with increasing annual number of OHCA patients at the destination hospital, especially in OHCA patients who experienced ROSC prior to arrival at the ED.

For a long time, previous studies examining institutional volume and patient prognosis have failed to show consensus on various aspects of this association [10–18]. A recently published systematic review has shown that cardiac arrest centers may improve the prognosis of OHCA patients [18, 26], but it is inconclusive as to whether OHCA patients should be transported directly to a cardiac arrest center [27, 28].

The divergent conclusions in previous studies may be attributed to differences in the target patient populations: some studies that reported no association between hospital volume and patient prognosis included patients with OHCA of non-cardiac origin [15, 16]. In contrast, the cohort of studies that reported improvement in patient outcomes at high-volume hospitals was restricted to patients with OHCA of cardiac origin [12–14]. Patients whose transport time was 10 min or less [12] and those with a shockable rhythm [13] were reported to have a better prognosis. Therefore, previous studies suggested that patients who are likely to survive and have a favorable neurological outcome are more likely to benefit from hospital volume. In the present study, we found a significant improvement in the neurological outcome of hospital volume when limited to patients with ROSC before arrival at the ED (Fig. 2). This result is consistent with that of several previous studies.

Meanwhile, another large-scale study of patients with OHCA (whose sample size was comparable to the current study) reported that no correlation existed between hospital volume and prognosis [17]. There are several possible reasons for the lack of a significant correlation between the size of the hospital and patient prognosis. Hospital factors such as location (urban/rural), teaching status, and 24-h cardiac interventional services have been reported to be correlated with prognosis [11, 14]. Similar studies have reported that physical volume, and nurses and rehabilitation therapists affect patient prognosis [29–33], although these studies did not investigate patients with cardiac arrest.

In addition, E-CPR for out-of-hospital cardiac arrest has been reported to be effective and not, and is an area of discussion [34–36]. In this study, most facilities were able to provide E-CPR regardless of hospital volume, and the percentage of patients who actually received E-CPR ranged from 14.8 to 26.0%. The results of the subgroup analysis of this study did not demonstrate any benefits of transport to a hospital to be able to provide E-CPR.

Additionally, previous analysis suggested that the treatment effect of cardiac arrest centers may be significantly better for patients with shockable rhythm and without prehospital ROSC [26]. The results of this study indicate that patients with prehospital ROSC are more likely to benefit from high-volume hospitals, although there was no significant difference between institutional volume and patient outcomes in patients without prehospital ROSC. This result may be a rationale for transferring patients with prehospital ROSC to high-volume hospitals, and that patients without ROSC do not benefit from being transported to high volume hospitals. This contradicts the results of previous study [26], and the characteristics of patients who should be transferred to high-volume hospitals and cardiac arrest centers is a topic for future study.

## Limitations

Although the sample size of this study was large, there was a bias in the number of patients in the groups, which was unavoidable owning to categorization, and may have affected the results. In this study, the number of patients included in the analysis was reduced from 34,754 to 3632, which has the effect of selection bias and survivor bias. This study was also limited to registry-participating hospitals, which resulted in a facility selection bias. This is because hospitals that participate in registries are more likely to be highly active. Moreover, there may be differences in the registration methods and omissions in registration depending on the hospital, which may affect the results. Limited information was available on the differences between hospitals and their respective characteristics. Hospitals characteristics besides the number of OHCA patients accepted may have affected the results, because we did not utilize factors related to hospitals as covariates in the generalized estimating equations.

The current study showed that the annual number of OHCA of cardiac origin patients admitted to a hospital may have a positive impact on favorable neurological prognosis, although other hospital characteristics were not considered. Further research is needed to identify the hospital characteristics with the optimal effect on OHCA patients.

## Conclusions

The annual number of OHCA patients received by the hospital did not significantly affect the prognosis of adult OHCA patients in most cases, although it was beneficial in cardiac arrest patients who achieved ROSC before arrival at the hospital’s ED. Thus, transport to a high-volume hospital may improve prognosis.

## Supplementary Information

**Additional file 1: Supplemental Table 1.** Characteristics of patients with OHCA who achieved ROSC before arrival at the ED.

**Additional file 2: Supplemental Table 2.** Characteristics of patients with OHCA who were transported to a critical-care medical center.

**Additional file 3: Supplemental Table 3.** Characteristics of patients with OHCA transported to ECMO-capable hospitals.

**Additional file 4: Supplemental Table 4.** Characteristics of patients with OHCA who did not achieve ROSC before arrival at the ED.

## Data Availability

The datasets generated and/or analysed during the current study are not publicly available due to the large amount of data but are available from the corresponding author on reasonable request.

## Abbreviations

CI: Confidence interval
CPC: Cerebral performance category
CPR: Cardiopulmonary resuscitation
ECG: Electrocardiogram
E-CPR: Extracorporeal cardiopulmonary resuscitation
ECMO: Extra corporeal membrane oxygenation
ED: Emergency department
EMS: Emergency medical services
GCS: Glasgow coma scale
ICU: Intensive care unit
JAAM-OHCA: Japanese Association for Acute Medicine out-of-hospital cardiac arrest
OHCA: Out-of-hospital cardiac arrest
OR: Odds ratio
ROSC: Return of spontaneous circulation
VA-ECMO: Veno-arterial extra corporeal membrane oxygenation
