# Supplementary material for: Effect of annual hospital admissions of out-of-hospital cardiac arrest patients on prognosis following cardiac arrest
Source: BMC Emerg Med. 2022 Jul 7;22:121. doi: 10.1186/s12873-022-00685-7 (PMC9261001; doi:10.1186/s12873-022-00685-7)
Supplement: Supplementary file 1 — Additional file 1: Supplemental Table 1. Characteristics of patients with OHCA who achieved ROSC before arrival at the ED. [file 12873_2022_685_MOESM1_ESM.docx]

Supplemental Table 1. Characteristics of patients with OHCA who achieved ROSC before arrival at the ED

|  | Low-volume  hospital | Medium-volume  hospital | High-volume  hospital |
| --- | --- | --- | --- |
| Institutions, n | 24 | 27 | 28 |
| Patients, n | 65 | 239 | 722 |
| Male, n (%) | 48 (73.8%) | 181 (75.7%) | 535 (74.1%) |
| Age, year | 68.0 (58.0–79.0) | 70.0 (58.5–80.0) | 69.0 (55.0–78.0) |
| Cause of cardiac OHCA, n (%) |  |  |  |
| Acute coronary syndrome | 26 (40.0%) | 89 (37.2%) | 248 (34.3%) |
| Other cardiac ^1^ | 23 (35.4%) | 83 (34.7%) | 223 (30.9%) |
| Presumed cardiac | 16 (24.6%) | 67 (28.0%) | 251 (34.8%) |
| Witness by bystander, n (%) | 55 (84.6%) | 196 (82.0%) | 534 (74.0%) |
| CPR initiated by bystander, n (%) | 39 (60.0%) | 114 (47.7%) | 389 (53.9%) |
| Defibrillation by bystander, n (%) | 1 (1.5%) | 20 (8.4%) | 70 (9.7%) |
| Primary ECG rhythm at the scene, n (%) |  |  |  |
| Ventricular fibrillation | 42 (64.6%) | 135 (56.5%) | 391 (54.2%) |
| Pulseless ventricular tachycardia | 0 (0.0%) | 8 (3.3%) | 9 (1.2%) |
| Pulseless electrical activity | 13 (20.0%) | 59 (24.7%) | 180 (24.9%) |
| Asystole | 10 (15.4%) | 37 (15.5%) | 142 (19.7%) |
| Treatments by EMS |  |  |  |
| Defibrillation, n (%) | 6 (9.2%) | 17 (7.1%) | 60 (8.3%) |
| Use of airway devices, n (%) | | | |
| Bag valve mask | 50 (76.9%) | 167 (69.9%) | 349 (48.3%) |
| Laryngeal mask airway | 2 (3.1%) | 2 (0.8%) | 54 (7.5%) |
| Esophageal obturator airway | 13 (20.0%) | 61 (25.5%) | 225 (31.2%) |
| Tracheal intubation | 0 (0.0%) | 9 (3.8%) | 94 (13.0%) |
| Intravenous fluid administration, n (%) | 19 (29.2%) | 108 (45.2%) | 339 (47.0%) |
| Treatments by doctor before arrival at ED, n (%) | 3 (4.6%) | 32 (13.4%) | 132 (18.3%) |
| Adrenaline dosage until arrival at ED (mg) | 1.0 (1.0–2.5) | 1.0 (1.0–2.0) | 2.0 (1.0–3.0) |
| Time (min.) |  |  |  |
| From calling EMS to arrival at the scene (min) | 8.0 (7.0–11.0) | 8.0 (6.0– 9.0) | 8.0 (6.0– 9.0) |
| From arrival at the scene to arrival at the ED (min) | 20.0 (16.0–29.0) | 23.0 (19.0–31.0) | 25.0 (19.0–32.0) |
| ECG rhythm on arrival at ED, n (%) | | | |
| Ventricular fibrillation | 0 (0.0%) | 0 (0.0%) | 0 (0.0%) |
| Pulseless ventricular tachycardia | 0 (0.0%) | 0 (0.0%) | 0 (0.0%) |
| Pulseless electrical activity | 0 (0.0%) | 0 (0.0%) | 0 (0.0%) |
| Asystole | 0 (0.0%) | 0 (0.0%) | 0 (0.0%) |
| Return of spontaneous circulation | 65 (100.0%) | 239 (100.0%) | 722 (100.0%) |
| Extracorporeal CPR, n (%) | 5 (7.7%) | 5 (2.1%) | 32 (4.4%) |
| Time from arrival at ED to start of VA ECMO (min) | 41.0 (32.2–72.2) | 132.0 (92.0–138.0) | 55.0 (30.5–109.5) |
| Laboratory data on arrival at the ED | | | |
| Serum urea nitrogen (mg/dL) | 18.9 (13.9–24.6) | 18.5 (15.0–23.5) | 18.0 (14.0–24.0) |
| Serum creatinine (mg/dL) | 1.07 (0.90–1.31) | 1.00 (0.83–1.30) | 1.02 (0.90–1.30) |
| Serum total protein (g/dL) | 6.6 (5.7–7.0) | 6.5 (6.2–7.0) | 6.4 (5.9–6.8) |
| Serum albumin (g/dL) | 3.8 (3.3–4.1) | 3.8 (3.4–4.1) | 3.6 (3.1–3.9) |
| pH | 7.17 (6.99–7.30) | 7.22 (7.01–7.31) | 7.19 (6.97–7.30) |
| PaCO_2_ (mmHg) | 46.5 (37.1–65.8) | 44.65 (36.0–64.9) | 46.1 (35.6–71.7) |
| PaO_2_ (mmHg) | 136.0 (84.4–283.6) | 132.0 (81.4–257.5) | 145.9 (78.3–292.3) |
| HCO_3_ (mEq/L) | 16.4 (14.00–19.4) | 17.5 (13.9–20.9) | 17.0 (13.8–19.8) |
| Base excess (mEq/L) | -11.6 (-17.0–-5.9) | -9.9 (-16.3–-5.0) | -11.2 (-15.9–-7.1) |
| Lactate (mg/dL) | 81.0 (57.9–102.6) | 70.5 (41.3–104.4) | 76.5 (55.8–104.4) |
| Glucose (mg/dL) | 233.0 (171.0–277.0) | 244.0 (194.0–297.0) | 246.00 (195.0–304.0) |
| Time from calling EMS to the first ROSC before arriving at the ED (min) | 18.0 (15.0–25.0) | 17.0 (12.0–23.0) | 17.0 (13.0–24.0) |
| Motor score of GCS in ED | 1.0 (1.0–4.0) | 1.0 (1.0–3.5) | 1.0 (1.0–2.0) |
| Therapeutic hypothermia, n (%) | 23 (35.4%) | 105 (43.9%) | 338 (46.8%) |
| Outcomes one month after cardiac arrest | | | |
| Survive, n (%) | 45 (69.2%) | 171 (71.5%) | 493 (68.3%) |
| Favorable neurological outcome, n (%) | 32 (49.2%) | 127 (53.1%) | 371 (51.4%) |

Data are presented as the median (25^th^–75^th^ percentile), percentage, or numbers.

^1^ “Other cardiac” causes include heart failure, valvular disease, cardiomyopathy, and cardiac diseases other than identified acute coronary syndrome.

OHCA: out-of-hospital cardiac arrest, CPR: cardiopulmonary resuscitation, EMS: emergency medical services, ECG: electrocardiogram, GCS: Glasgow coma scale, VA ECMO: veno-arterial extra corporeal membrane oxygenation, ED: emergency department, ROSC: return of spontaneous circulation.
