# Supplementary material for: Effect of annual hospital admissions of out-of-hospital cardiac arrest patients on prognosis following cardiac arrest
Source: BMC Emerg Med. 2022 Jul 7;22:121. doi: 10.1186/s12873-022-00685-7 (PMC9261001; doi:10.1186/s12873-022-00685-7)
Supplement: Supplementary file 2 — Additional file 2: Supplemental Table 2. Characteristics of patients with OHCA who were transported to a critical-care medical center. [file 12873_2022_685_MOESM2_ESM.docx]

Supplemental Table 2. Characteristics of patients with OHCA who were transported to a critical-care medical center

|  | Low-volume  hospital | Medium-volume  hospital | High-volume  hospital |
| --- | --- | --- | --- |
| Institutions, n | 16 | 21 | 29 |
| Patients, n | 167 | 585 | 2,565 |
| Male, n (%) | 112 (67.1%) | 429 (73.3%) | 1,852 (72.2%) |
| Age, year | 69.0 (58.0–81.0) | 69.0 (59.0–80.0) | 69.0 (58.0–79.0) |
| Cause of OHCA, n (%) |  |  |  |
| Acute coronary syndrome | 52 (31.1%) | 209 (35.7%) | 805 (31.4%) |
| Other cardiac ^1^ | 51 (30.5%) | 177 (30.3%) | 620 (24.2%) |
| Presumed cardiac | 64 (38.3%) | 199 (34.0%) | 1,140 (44.4%) |
| Bystander-witnessed cardiac arrest, n (%) | 114 (68.3%) | 420 (71.8%) | 1,754 (68.4%) |
| CPR initiated by bystander, n (%) | 85 (50.9%) | 253 (43.2%) | 1,233 (48.1%) |
| Defibrillation by bystander, n (%) | 5 (3.0%) | 38 (6.5%) | 160 (6.2%) |
| Primary ECG rhythm at the scene, n (%) |  |  |  |
| Ventricular fibrillation | 64 (38.3%) | 262 (44.8%) | 1,113 (43.4%) |
| Pulseless ventricular tachycardia | 1 (0.6%) | 9 (1.5%) | 16 (0.6%) |
| Pulseless electrical activity | 45 (26.9%) | 156 (26.7%) | 701 (27.3%) |
| Asystole | 57 (34.1%) | 158 (27.0%) | 735 (28.7%) |
| Treatments by EMS |  |  |  |
| Defibrillation, n (%) | 46 (27.5%) | 154 (26.3%) | 722 (28.1%) |
| Use of airway devices, n (%) | | | |
| Bag valve mask | 131 (78.4%) | 354 (60.5%) | 1,041 (40.6%) |
| Laryngeal mask airway | 2 (1.2%) | 20 (3.4%) | 171 (6.7%) |
| Esophageal obturator airway | 26 (15.6%) | 179 (30.6%) | 940 (36.6%) |
| Tracheal intubation | 8 (4.8%) | 32 (5.5%) | 413 (16.1%) |
| Intravenous fluid administration, n (%) | 47 (28.1%) | 249 (42.6%) | 1,120 (43.7%) |
| Treatments by doctor before arrival at ED, n (%) | 14 (8.4%) | 84 (14.4%) | 566 (22.1%) |
| Adrenaline dosage until arrival at ED (mg) | 3.0 (1.5–5.0) | 3.0 (2.0–5.0) | 2.0 (1.0–4.0) |
| Time (min) |  |  |  |
| From calling EMS to arrival at the scene (min) | 9.0 (7.0–11.0) | 8.0 (7.0–10.0) | 8.0 (6.0–10.0) |
| From arrival at the scene to arrival at the ED (min) | 21.0 (17.0–28.5) | 23.0 (18.0–30.0) | 24.0 (18.0–31.0) |
| ECG rhythm on arrival at ED, n (%) | | | |
| Ventricular fibrillation | 22 (13.2%) | 77(13.2%) | 469(18.3%) |
| Pulseless ventricular tachycardia | 2 (1.2%) | 4(0.7%) | 15(0.6%) |
| Pulseless electrical activity | 45 (26.9%) | 154(26.3%) | 645(25.1%) |
| Asystole | 55 (32.9%) | 156(26.7%) | 714(27.8%) |
| Return of spontaneous circulation | 43 (25.7%) | 194(33.2%) | 722(28.1%) |
| Extracorporeal CPR, n (%) | 30 (18.0%) | 113 (19.3%) | 667 (26.0%) |
| Time from arrival at ED to start of VA ECMO (min) | 35.0 (26.0–62.0) | 38.0 (26.0–63.0) | 29.0 (20.0–41.0) |
| Laboratory data on arrival at the ED | | | |
| Serum urea nitrogen (mg/dL) | 18.7 (14.0–29.0) | 19.0 (15.0–26.0) | 18.9 (14.0–26.6) |
| Serum creatinine (mg/dL) | 1.14 (0.90–1.51) | 1.14 (0.91–1.40) | 1.11 (0.90–1.50) |
| Serum total protein (g/dL) | 6.2 (5.7–6.7) | 6.1 (5.5–6.6) | 6.1 (5.4–6.6) |
| Serum albumin (g/dL) | 3.4 (3.0–3.7) | 3.4 (2.9–3.8) | 3.3 (2.8–3.7) |
| pH | 7.08 (6.93–7.27) | 7.10 (6.94–7.26) | 7.06 (6.90–7.25) |
| PaCO_2_ (mmHg) | 48.7 (37.4–71.8) | 52.0 (39.7–73.4) | 51.8 (37.3–77.5) |
| PaO_2_ (mmHg) | 154.8 (83.2–283.6) | 137.0 (79.0–280.0) | 169.0 (82.6–340.0) |
| HCO_3_ (mEq/L) | 14.9 (12.2–18.9) | 16.4 (11.9–20.1) | 15.4 (11.9–18.8) |
| Base excess (mEq/L) | -13.9 (-17.9–-7.9) | -12.6 (-18.4–-6.6) | -14.4 (-20.2–-8.6) |
| Lactate (mg/dL) | 104.7 (76.0–130.1) | 90.0 (56.0–122.4) | 95.0 (65.7–128.7) |
| Glucose (mg/dL) | 259.5 (179.0–302.3) | 270.5 (206.3–334.0) | 263.0 (199.0–330.0) |
| Patient with ROSC prior to arrival at ED, n (%) | 42 (25.1%) | 185 (31.6%) | 696 (27.1%) |
| Time from calling EMS to the first ROSC before arriving at the ED (min) ^2^ | 21.0 (14.0–26.0) | 18.0 (13.0–24.0) | 19.0 (13.0–26.0) |
| Time from calling EMS to the first ROSC after arriving at the ED (min) ^3^ | 43.0 (33.0–57.3) | 43.0 (35.0–58.0) | 44.0 (34.0–57.0) |
| Time from ED arrival to ROSC after admission (min) ^3^ | 13.0 (8.0–18.2) | 13.0 (8.0–22.2) | 13.0 (8.0–24.0) |
| Motor score of GCS in ED | 1.0 (1.0–1.0) | 1.0 (1.0–1.0) | 1.0 (1.0–1.0) |
| Therapeutic hypothermia, n (%) | 52 (31.1%) | 203 (34.7%) | 955 (37.2%) |
| Outcomes one month after cardiac arrest | | | |
| Survive, n (%) | 57 (34.1%) | 224 (38.3%) | 883 (34.4%) |
| Favorable neurological outcome, n (%) | 32 (19.2%) | 145 (24.8%) | 543 (21.2%) |

Data are presented as the median (25^th^–75^th^ percentile), percentage, or numbers.

^1^ “Other cardiac” causes include heart failure, valvular disease, cardiomyopathy, and cardiac diseases other than identified acute coronary syndrome.

^2^ Data limited to cases with ROSC prior to ED arrival.

^3^ Data limited to cases with cardiac arrest on arrival at the ED.

OHCA: out-of-hospital cardiac arrest, CPR: cardiopulmonary resuscitation, EMS: emergency medical services, ECG: electrocardiogram, GCS: Glasgow coma scale, VA ECMO: veno-arterial extra corporeal membrane oxygenation, ED: emergency department, ROSC: return of spontaneous circulation.
