# Supplementary material for: Effect of annual hospital admissions of out-of-hospital cardiac arrest patients on prognosis following cardiac arrest
Source: BMC Emerg Med. 2022 Jul 7;22:121. doi: 10.1186/s12873-022-00685-7 (PMC9261001; doi:10.1186/s12873-022-00685-7)
Supplement: Supplementary file 3 — Additional file 3: Supplemental Table 3. Characteristics of patients with OHCA transported to ECMO-capable hospitals. [file 12873_2022_685_MOESM3_ESM.docx]

Supplemental Table 3. Characteristics of patients with OHCA transported to ECMO-capable hospitals

|  | Low-volume  hospital | Medium-volume  hospital | High-volume  hospital |
| --- | --- | --- | --- |
| Institutions, n | 26 | 27 | 29 |
| Patients, n | 222 | 777 | 2,565 |
| Male, n (%) | 151 (68.0%) | 551 (70.9%) | 1,852 (72.2%) |
| Age, year | 70.0 (58.0–82.0) | 71.0 (60.0–82.0) | 69.0 (58.0–79.0) |
| Cause of OHCA, n (%) |  |  |  |
| Acute coronary syndrome | 68 (30.6%) | 260 (33.5%) | 805 (31.4%) |
| Other cardiac ^1^ | 65 (29.3%) | 222 (28.6%) | 620 (24.2%) |
| Presumed cardiac | 89 (40.1%) | 295 (38.0%) | 1,140 (44.4%) |
| Bystander-witnessed cardiac arrest, n (%) | 155 (69.8%) | 533 (68.6%) | 1,754 (68.4%) |
| CPR initiated by bystander, n (%) | 116 (52.3%) | 345 (44.4%) | 1,233 (48.1%) |
| Defibrillation by bystander, n (%) | 5 (2.3%) | 46 (5.9%) | 160 (6.2%) |
| Primary ECG rhythm at the scene, n (%) |  |  |  |
| Ventricular fibrillation | 81 (36.5%) | 312 (40.2%) | 1,113 (43.4%) |
| Pulseless ventricular tachycardia | 1 (0.5%) | 10 (1.3%) | 16 (0.6%) |
| Pulseless electrical activity | 62 (27.9%) | 208 (26.8%) | 701 (27.3%) |
| Asystole | 78 (35.1%) | 247 (31.8%) | 735 (28.7%) |
| Treatments by EMS |  |  |  |
| Defibrillation, n (%) | 53 (23.9%) | 193 (24.8%) | 722 (28.1%) |
| Use of airway devices, n (%) | | | |
| Bag valve mask | 169 (76.1%) | 463 (59.6%) | 1,041 (40.6%) |
| Laryngeal mask airway | 5 (2.3%) | 21 (2.7%) | 171 (6.7%) |
| Esophageal obturator airway | 35 (15.8%) | 250 (32.2%) | 940 (36.6%) |
| Tracheal intubation | 13 (5.9%) | 43 (5.5%) | 413 (16.1%) |
| Intravenous fluid administration, n (%) | 64 (28.8%) | 313 (40.3%) | 1,120 (43.7%) |
| Treatments by doctor before arrival at ED, n (%) | 22 (9.9%) | 85 (10.9%) | 566 (22.1%) |
| Adrenaline dosage until arrival at ED (mg) | 3.0 (1.0–4.0) | 3.0 (2.0–5.0) | 2.0 (1.0–4.0) |
| Time (min) |  |  |  |
| From calling EMS to arrival at the scene (min) | 9.0 (7.0–11.0) | 8.0 (6.0–10.0) | 8.0 (6.0–10.0) |
| From arrival at the scene to arrival at the ED (min) | 22.0 (17.0–29.0) | 22.0 (17.0–29.0) | 24.0 (18.0–31.0) |
| ECG rhythm on arrival at ED, n (%) | | | |
| Ventricular fibrillation | 25 (11.3%) | 100 (12.9%) | 469 (18.3%) |
| Pulseless ventricular tachycardia | 4 (1.8%) | 8 (1.0%) | 15 (0.6%) |
| Pulseless electrical activity | 60 (27.0%) | 208 (26.8%) | 645 (25.1%) |
| Asystole | 77 (34.7%) | 234 (30.1%) | 714 (27.8%) |
| Return of spontaneous circulation | 56 (25.2%) | 227 (29.2%) | 722 (28.1%) |
| Extracorporeal CPR, n (%) | 35 (15.8%) | 139 (17.9%) | 667 (26.0%) |
| Time from arrival at ED to start of VA ECMO (min) | 35.5 (27.0–58.5) | 41.0 (28.5–64.5) | 29.0 (20.0–41.0) |
| Laboratory data on arrival at the ED | | | |
| Serum urea nitrogen (mg/dL) | 19.1 (14.0–31.3) | 19.0 (14.9–27.0) | 18.9 (14.0–26.6) |
| Serum creatinine (mg/dL) | 1.17 (0.95–1.66) | 1.13 (0.90–1.42) | 1.11 (0.90–1.50) |
| Serum total protein (g/dL) | 6.2 (5.7–6.7) | 6.1 (5.5–6.7) | 6.1 (5.4–6.6) |
| Serum albumin (g/dL) | 3.4 (3.0–3.8) | 3.4 (2.9–3.8) | 3.3 (2.8–3.7) |
| pH | 7.07 (6.92–7.25) | 7.10 (6.93–7.25) | 7.06 (6.90–7.25) |
| PaCO_2_ (mmHg) | 50.8 (38.1–77.1) | 53.0 (39.1–73.5) | 51.8 (37.3–77.5) |
| PaO_2_ (mmHg) | 148.5 (82.6–306.5) | 137.0 (81.3–279.1) | 169.0 (82.6–340.0) |
| HCO_3_ (mEq/L) | 16.1 (12.7–19.5) | 16.3 (12.0–19.9) | 15.4 (11.9–18.8) |
| Base excess (mEq/L) | -13.5 (-18.0–-7.9) | -12.9 (-18.7–-6.9) | -14.4 (-20.2–-8.6) |
| Lactate (mg/dL) | 102.6 (72.5–130.5) | 91.0 (59.0–123.0) | 95.0 (65.7–128.7) |
| Glucose (mg/dL) | 243.0 (170.3–299.5) | 261.5 (198.8–331.3) | 263.0 (199.0–330.0) |
| Patient with ROSC prior to arrival at ED, n (%) | 55 (24.8%) | 212 (27.3%) | 696 (27.1%) |
| Time from calling EMS to the first ROSC before arriving at the ED (min) ^2^ | 22.0 (15.0–26.0) | 18.0 (13.0–25.0) | 19.0 (13.0–26.0) |
| Time from calling EMS to the first ROSC after arriving at the ED (min) ^3^ | 44.0 (35.0–57.0) | 43.0 (34.0–57.0) | 44.0 (34.0–57.0) |
| Time from ED arrival to ROSC after admission (min) ^3^ | 13.0 (8.0–20.0) | 13.0 (8.0–23.0) | 13.0 (8.0–24.0) |
| Motor score of GCS in ED | 1.0 (1.0–1.0) | 1.0 (1.0–1.0) | 1.0(1.0–1.0) |
| Therapeutic hypothermia, n (%) | 71 (32.0%) | 241 (31.0%) | 955 (37.2%) |
| Outcomes one month after cardiac arrest | | | |
| Survive, n (%) | 76 (34.2%) | 260 (33.5%) | 883 (34.4%) |
| Favorable neurological outcome, n (%) | 42 (18.9%) | 160 (20.6%) | 543 (21.2%) |

Data are presented as the median (25^th^-75^th^ percentile), percentage, or numbers.

^1^ “Other cardiac” causes include heart failure, valvular disease, cardiomyopathy, and cardiac diseases other than identified acute coronary syndrome.

^2^ Data limited to cases with ROSC prior to ED arrival.

^3^ Data limited to cases with cardiac arrest on arrival at the ED.

OHCA: out-of-hospital cardiac arrest, CPR: cardiopulmonary resuscitation, EMS: emergency medical services, ECG: electrocardiogram, GCS: Glasgow coma scale, VA ECMO: veno-arterial extra corporeal membrane oxygenation, ED: emergency department, ROSC: return of spontaneous circulation.
