# Supplementary material for: Effect of annual hospital admissions of out-of-hospital cardiac arrest patients on prognosis following cardiac arrest
Source: BMC Emerg Med. 2022 Jul 7;22:121. doi: 10.1186/s12873-022-00685-7 (PMC9261001; doi:10.1186/s12873-022-00685-7)
Supplement: Supplementary file 4 — Additional file 4: Supplemental Table 4. Characteristics of patients with OHCA who did not achieve ROSC before arrival at the ED. [file 12873_2022_685_MOESM4_ESM.docx]

Supplemental Table 4. Characteristics of patients with OHCA who did not achieve ROSC before arrival at the ED

|  | Low-volume  hospital | Medium-volume  hospital | High-volume  hospital |
| --- | --- | --- | --- |
| Institutions, n | 29 | 28 | 29 |
| Patients, n | 185 | 578 | 1843 |
| Male, n (%) | 122 (65.9%) | 394 (68.2%) | 1317 (71.5%) |
| Age, year | 71.0 (62.0–84.0) | 72.0 (62.0–82.0) | 69.0 (58.0–80.0) |
| Cause of cardiac OHCA, n (%) |  |  |  |
| Acute coronary syndrome | 51 (27.6%) | 180 (31.1%) | 557 (30.2%) |
| Other cardiac ^1^ | 52 (28.1%) | 143 (24.7%) | 397 (21.5%) |
| Presumed cardiac | 82 (44.3%) | 255 (44.1%) | 889 (48.2%) |
| Witness by bystander, n (%) | 116 (62.7%) | 368 (63.7%) | 1220 (66.2%) |
| CPR initiated by bystander, n (%) | 91 (49.2%) | 243 (42.0%) | 839 (45.5%) |
| Defibrillation by bystander, n (%) | 4 (2.2%) | 28 (4.8%) | 90 (4.9%) |
| Primary ECG rhythm at the scene, n (%) |  |  |  |
| Ventricular fibrillation | 47 (25.4%) | 190 (32.9%) | 722 (39.2%) |
| Pulseless ventricular tachycardia | 1 (0.5%) | 2 (0.3%) | 7 (0.4%) |
| Pulseless electrical activity | 58 (31.4%) | 164 (28.4%) | 521 (28.3%) |
| Asystole | 79 (42.7%) | 222 (38.4%) | 593 (32.2%) |
| Treatments by EMS |  |  |  |
| Defibrillation, n (%) | 52 (28.1%) | 179 (31.0%) | 662 (35.9%) |
| Use of airway devices, n (%) | | | |
| Bag valve mask | 129 (69.7%) | 318 (55.0%) | 692 (37.5%) |
| Laryngeal mask airway | 3 (1.6%) | 19 (3.3%) | 117 (6.3%) |
| Esophageal obturator airway | 40 (21.6%) | 206 (35.6%) | 715 (38.8%) |
| Tracheal intubation | 13 (7.0%) | 35 (6.1%) | 319 (17.3%) |
| Intravenous fluid administration, n (%) | 59 (32.4%) | 221 (38.2%) | 781 (42.4%) |
| Treatments by doctor before arrival at ED, n (%) | 20 (10.8%) | 59 (10.2%) | 434 (23.5%) |
| Adrenaline dosage until arrival at ED (mg) | 3.0 (2.0–4.0) | 3.0 (2.0–5.0) | 3.0 (1.0–4.0) |
| Time (min.) |  |  |  |
| From calling EMS to arrival at the scene (min) | 8.0 (7.0–11.0) | 8.0 (6.0– 9.0) | 8.0 (6.0– 9.0) |
| From arrival at the scene to arrival at the ED (min) | 20.0 (16.0–29.0) | 23.0 (19.0–31.0) | 25.0 (19.0–32.0) |
| ECG rhythm on arrival at ED, n (%) | | | |
| Ventricular fibrillation | 28 (15.1%) | 101 (17.5%) | 469 (25.4%) |
| Pulseless ventricular tachycardia | 5 (2.7%) | 8 (1.4%) | 15 (0.8%) |
| Pulseless electrical activity | 65 (35.1%) | 220 (38.1%) | 645 (35.0%) |
| Asystole | 87 (47.0%) | 249 (43.1%) | 714 (38.7%) |
| Return of spontaneous circulation | 0 (0.0%) | 0 (0.0%) | 0 (0.0%) |
| Extracorporeal CPR, n (%) | 32 (17.3%) | 139 (24.0%) | 635 (34.5%) |
| Time from arrival at ED to start of VA ECMO (min) | 35.5 (26.0–63.0) | 39.0 (29.0–63.0) | 28.5 (20.0–40.0) |
| Laboratory data on arrival at the ED | | | |
| Serum urea nitrogen (mg/dL) | 20.0 (14.8–32.8) | 19.4 (14.8–29.0) | 19.0 (14.0–28.0) |
| Serum creatinine (mg/dL) | 1.19 (0.96–1.69) | 1.20 (0.92–1.50) | 1.18 (0.91–1.60) |
| Serum total protein (g/dL) | 6.2 (5.7–6.7) | 6.0 (5.3–6.4) | 5.9 (5.3–6.5) |
| Serum albumin (g/dL) | 3.3 (2.9–3.7) | 3.2 (2.7–3.6) | 3.1 (2.6–3.5) |
| pH | 7.03 (6.89–7.25) | 7.05 (6.90–7.20) | 7.01 (6.87–7.20) |
| PaCO_2_ (mmHg) | 57.4 (39.9–80.1) | 57.0 (41.7–75.7) | 55.7 (39.4–80.4) |
| PaO_2_ (mmHg) | 148.5 (82.1–295.4) | 142.8 (81.4–301.0) | 181.2 (85.8–361.8) |
| HCO_3_ (mEq/L) | 16.0 (12.1–20.2) | 15.2 (11.5–18.7) | 14.4 (10.9–17.9) |
| Base excess (mEq/L) | -14.2 (-18.1– -8.1) | -14.1 (-20.0– -8.5) | -16.2 (-21.6– -10.4) |
| Lactate (mg/dL) | 108.5 (78.3–141.8) | 100.8 (73.0–129.0) | 108.0 (78.1–141.8) |
| Glucose (mg/dL) | 244.0 (173.0–307.0) | 274.0 (203.0–349.0) | 278.0 (203.0–346.0) |
| Motor score of GCS in ED | 1.0 (1.0–1.0) | 1.0 (1.0–1.0) | 1.0 (1.0–1.0) |
| Therapeutic hypothermia, n (%) | 51 (27.6%) | 151 (26.1%) | 617 (33.5%) |
| Outcomes one month after cardiac arrest | | | |
| Survive, n (%) | 36 (19.5%) | 98 (17.0%) | 390 (21.2%) |
| Favorable neurological outcome, n (%) | 14 (7.6%) | 40 (6.9%) | 172 (9.3%) |

Data are presented as the median (25^th^–75^th^ percentile), percentage, or numbers.

^1^ “Other cardiac” causes include heart failure, valvular disease, cardiomyopathy, and cardiac diseases other than identified acute coronary syndrome.

OHCA: out–of–hospital cardiac arrest, CPR: cardiopulmonary resuscitation, EMS: emergency medical services, ECG: electrocardiogram, GCS: Glasgow coma scale, VA ECMO: veno–arterial extra corporeal membrane oxygenation, ED: emergency department, ROSC: return of spontaneous circulation.
